# Clioquinol improves motor and non-motor deficits in MPTP-induced monkey model of Parkinson’s disease through AKT/mTOR pathway

**DOI:** 10.18632/aging.103225

**Published:** 2020-05-18

**Authors:** Liangqin Shi, Chao Huang, Qihui Luo, Yu Xia, Wentao Liu, Wen Zeng, Anchun Cheng, Riyi Shi, Chen Zhengli

**Affiliations:** 1 Laboratory of Animal Disease Model, College of Veterinary Medicine, Sichuan Agricultural University, Chengdu Sichuan, China; 2 Department of Basic Medical Sciences, College of Veterinary Medicine, Purdue University, West Lafayette, IN 47907, USA; 3 Key Laboratory of Animal Disease and Human Health of Sichuan Province, College of Veterinary Medicine, Sichuan Agricultural University, Ya’an, Sichuan, China; 4 Sichuan Primed Biological Technology Co., Ltd, National Experimental Macaque Reproduce Laboratory, Ya’an, Sichuan, China

**Keywords:** Parkinson’s disease, clioquinol, iron, ferroptosis, monkey

## Abstract

Despite decades of research into the pathology mechanisms of Parkinson’s disease (PD), disease-modifying therapy of PD is scarce. Thus, searching for new drugs or more effective neurosurgical treatments has elicited much interest. Clioquinol (CQ) has been shown to have therapeutic benefits in rodent models of neurodegenerative disorders. However, it’s neuroprotective role and mechanisms in PD primate models and PD patients, especially in the advanced stages, are not fully understood. Furthermore, issues such as spontaneous recovery of motor function and high symptom variability in different monkeys after the same toxic protocol, has not been resolved before the present study. In this study, we designed a chronic and long-term progressive protocol to generate a stabilized PD monkey model showed with classic motor and non-motor deficits, followed by treatment analysis of CQ. We found that CQ could remarkably improve the motor and non-motor deficits, which were based on the reduction of iron content and ROS level in the SN and further improvement in pathology. Meanwhile, we also showed that ferroptosis was probably involved in the pathogenesis of PD. In addition, the study shows a positive effect of CQ on AKT/mTOR survival pathway and a blocking effect on p53 medicated cell death *in vivo and in vitro.*

## INTRODUCTION

Parkinson's disease (PD), an age-dependent neurodegeneration disorder, affects about 1% of the elderly population in the world [1]. The pathology of PD is principally characterized by the loss of A9-type dopaminergic (DA) neurons in the substantia nigra pars compacta (SNpc), resulting in progressive loss of motor functions and other symptoms [2]. The disease-modifying therapy of PD is scarce, and the pathologic mechanisms of which remains unclear. Genetic mutations, either dominant (PARK3, LRRK2, UCH-L1, PARK13, and SNCA) or recessive (Parkin, PINK1, FBXO7, PLA2G6, and PARK9), are causally involved in rare familial PD, while environmental and cellular toxins are reported to contribute to sporadic PD [3–5]. Multiple pathogenic mechanisms, especially reactive oxygen species (ROS) accumulation, have been implicated in both rare familial and sporadic PD. ROS are common products generated during cellular processes. Moderate ROS levels are essential for cell function, while excessive ROS may be toxic to cells, resulting in damage to lipids, proteins and DNAs [6, 7]. The concept of free radical involvement is supported by increased levels of malondialdehyde, lipid hydroperoxides, protein and DNA oxidation products in the SN of PD patients, accompanied with decreased GSH levels [8–10]. However, the role of oxidative stress in PD is far from clear due to its complex production pathway.

Considerable damage occurs before the onset of clinical symptoms in PD patients, making identification of early events a challenge. Therefore, more animal disease models are needed and critical for PD studies, especially primate animal models. 1-methyl-4-phenyl-1,2,3,6-tetrahydropyridine (MPTP) injection is widely applicated for primate PD models. After injection, MPTP is metabolized in astrocytes to the active MPP^+^ molecule. DA neurons then selectively uptake MPP^+^, and death results from mitochondrial dysfunction related oxidative stress [11, 12]. The MPTP intoxication monkey model can reproduce not only the pathologic damages but also the behavioral symptoms, such as bradykinesia (slowness of movement), rigidity and tremors [13, 14]. However, several limitations of these models have limited its further applications. Many studies have detected spontaneous recovery from motor symptoms in MPTP monkey models, which is not observed in humans suffering from PD and may interfere with physiologic mechanism studies and the evaluation of therapeutic interventions [15, 16]. Meanwhile, variable severity of symptoms in different monkeys, difficulty in producing gradual death of DA neurons, and a progressive appearance of behavioral symptoms, makes the development of more stabilized monkey models of importance [17].

Enormous progress has been made in the treatment of PD, with levodopa (LD) remaining the most potent drug for controlling PD symptoms, especially those related to bradykinesia [18, 19]. But, acute side effects, such as nausea, vomiting, orthostatic hypotension and levodopa-induced dyskinesias, complicate this therapy. Yet, even these complications were lessened by the treatment of LD combined with a peripheral dopa-decarboxylase inhibitor (carbidopa or benserazide) [20–23]. Thus, searching for new drugs or neurosurgical treatments, which function more effective, has elicited much interest. Clioquinol (CQ) is an iodinated 8-hyxroyquinoline (8-HQ), which for decades has been widely used as an anti-parasitic agent [24], has emerging efficacy in arresting metal-mediated neurotoxicity in animal models of neurodegenerative disorders [25, 26]. Cherny et al. showed that CQ could inhibit β-Amyloid accumulation in Alzheimer's Disease transgenic mice [27], and Nguyen et al. found an improvement of behavioral and pathologic phenotypes in transgenic Huntington’s mice (R6/2) after CQ treatment [28]. Meanwhile, research has indicated that CQ can exhibited protection against MPTP induced neurotoxicity in mice [29], and improved cognitive, motor function, and other pathological features of PD transgenic mice (A53T) [30]. The mechanism involved in these findings may be redox-silencing of reactive Fe^2+^ or the prevention of fibrillization of the peptide [31, 32]. Considering its unclear mechanism of action and little study in PD primate models and PD subjects, the potential of CQ demands further study in the search for an effective PD treatment.

In this report, we developed a long-term progressive protocol according to individual sensitivity, in order to generate stabilized primate PD models induced by MPTP with rhesus monkeys. Our model can reproduce not only destruction of DA systems but also most behavioral symptoms, such as bradykinesia, dyskinesia, rigidity, tremor (especially resting tremor), freezing gait, crouched posture, accompanied with typically PD non-motor symptoms including dysphagia, hypersomnia, constipation, less vocalization, salivation, hyporeactive social interactions, and mask face. All these symptoms could be stabilized for more than 3 weeks without recovery after the withdrawal of MPTP. Then the evaluation of CQ on PD treatment was followed, where the LD was set as a positive control. We found that CQ treatment could remarkably improve the motor and no-motor deficits in PD monkey models after the chronic and long-term MPTP intoxication, which was based on improvement of pathology, suppression of iron content and ROS in the SN, and probably inhibition of ferroptosis. Furthermore, we found that CQ could activate AKT/mTOR signals and inhibit p-p53 in control of bcl-2 and bax in both *in vivo* and *in vitro* systems. Our work has generated stabilized monkey models of PD with motor and non-motor deficits, and has effectively demonstrated the efficacy of CQ in the treatment of PD, which will facilitate further the pathological studies and drug discovery.

## RESULTS

### Behavioral assessment: establishment of a chronic and progressive monkey model

**Figure 1:**
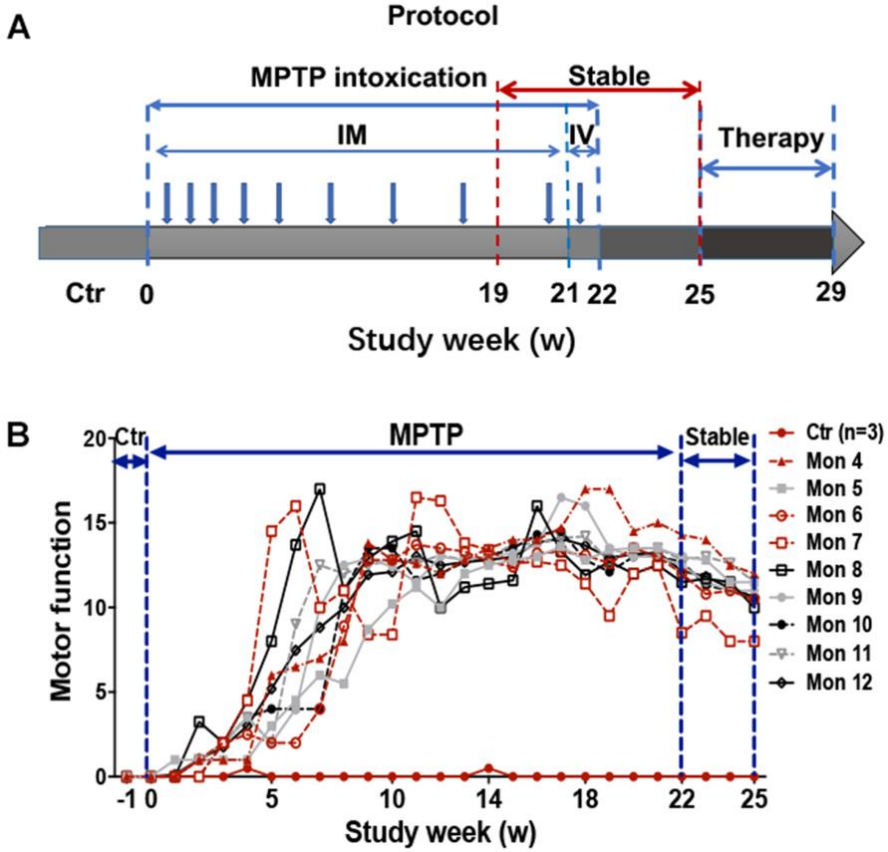
**Experimental design of the study and behavior test by Kurlan rating scale.** (**A**) A progressive intoxication protocol of MPTP from 0-22w, MPTP withdraw period from 22-25w, and treatment period from 25-29w. IM: Intramuscular injection; IV: Intravenous injection. (**B**) Longitudinal evaluation (in weeks) of motor function by Kurlan rating scale for each monkey during the MPTP intoxication and withdraw period.

Overall, monkeys undergoing chronic progressive intoxication received a cumulative dose (27.14 ± 12.41mg/kg) of MPTP for 22 weeks (Figure 1A), and exhibited tremor at the 35.63±8.77^th^ day and tremor stabled at the 74±24.8^th^ day (Table 1). Specifically, behavior tests revealed no apparent clinical symptoms in MPTP treated monkeys after the first three-week daily injection of MPTP (Figure 1B) when compared with control group. However, with continued MPTP injection, obvious motor abnormalities were observed in two of them at the 4^th^ week, and mild PD symptoms observed in all the monkeys at the 9^th^ week (Figure 1B), and the classic and stabled parkinsonism symptoms, including resting tremor, rigidity, bradykinesia, and postural instability, were gradually developed at the 18^th^ week, since then no significant variation in MPTP group monkeys were observed (Figure 1B). In addition, some non-motor disabilities, including hypersomnia, yawning, constipation, salivation, decreased self-care ability, decreased vocalization, decreased spontaneous eye activity, and decreased trigger eye activity, were also observed during the MPTP intoxication period. The behavior test result indicated that we generated a chronic and progressive monkey model of Parkinson’s disease that imitated almost all the motor and non-motor deficits of PD. After 3 weeks of withdraw period (from 22-25 week), the MPTP group monkeys were randomly divided into three subgroups, MPTP group, MPTP+LD group, and MPTP+CQ group. After 4 weeks of treatment, all the monkeys were sacrificed at the end of 29 week (Figure 1A).

**Table 1:** Information of MPTP administration and animal sensitivity analysis to MPTP.

**MPTP group**	**First tremor**		**Stable tremor**		**Total days of MPTP**	**Total days of model**	**Total dose of MPTP (mg/kg)**
	**Days**	**Dose of MPTP (mg/kg)**	**Days**	**Dose of MPTP (mg/kg)**			
Total	35.63±8.77	7.13±1.75	74±24.8	15.16±8.97	104.38±29.16	205	27.14±12.41
Sensitive (N=3)	27.33±1.70	5.47±0.34	51.00±1.41	7.13±0.75	74.00±4.08	205	15.83±1.47
Moderately sensitive (N=3)	34.67±2.05	6.93±0.41	78.67±24.07	16.1±8.30	106.33±10.62	205	25.17±3.38
Hyporesponsiveness (N=3)	49.5±0.5	9.9±0.1	101.5±2.5	25.8±2.5	147	205	47.05±1.35

### CQ improved motor and non-motor deficits after MPTP intoxication

**Figure 2:**
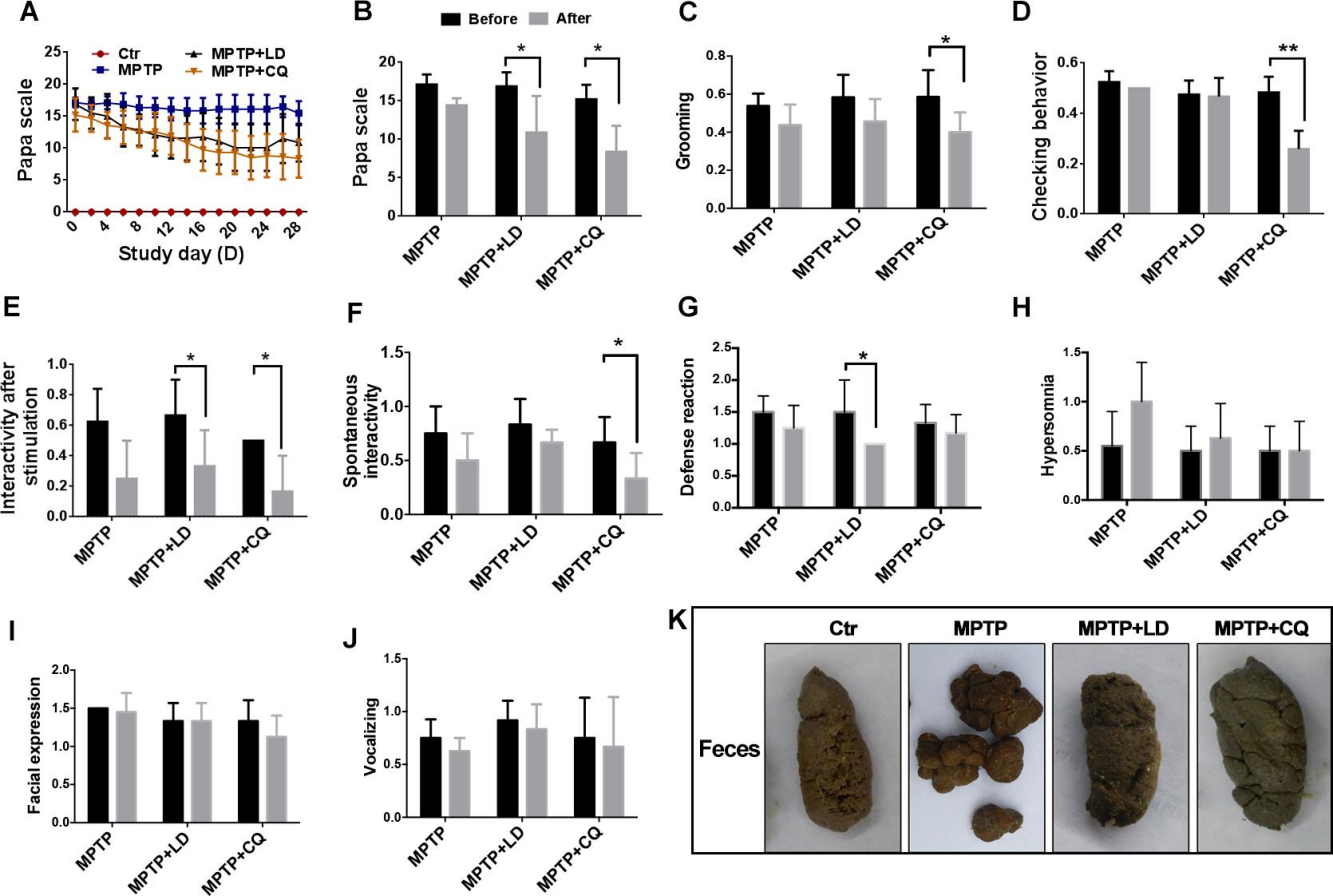
**CQ improved clinical phenotypes after long-term of MPTP intoxication.** (**A**) Evaluation of motor function for each group by Papa scale during the treatment period. Motor deficits mitigated by both LD and CQ treatment displayed with decreased Papa scale. (**B**) The comparison of Papa scores in each group before and after treatment. Papa scores were significantly declined by both LD and CQ treatment. (**C**–**J**) The comparison of spirit evaluation, including grooming score, checking behavior score, interactivity after stimulation score, spontaneous interactivity score, defense reaction score, hypersomnia score, facial expression score, and vocalizing score in each group, before and after treatment, respectively. (**K**) Representative images showed constipation after MPTP intoxication, and which was relieved by both LD and CQ treatment. Data expressed as the mean ± SD. *P<0.05, **P<0.01, indicate significant difference.

During the treatment period, Papa scale was carried out to evaluate the motor disabilities. At the beginning of the treatment, there was no significant difference of Papa scores between each group. Subsequently, with the progress of treatment developed, the clinical scores of both MPTP+LD group and MPTP+CQ group decreased gradually during the treatment period (Figure 2A), and significantly decreased (P<0.05) at the end of the treatment (the average score of the last two evaluations) when compared with before treatment (0 day) (Figure 2B). Specifically, posture, tremor, hand movements, climbing, holding food and social interactions scores of MPTP+LD group were significantly decreased (P<0.05 or P<0.01) when compared with pre-treatment. Tremor, general mobility, hand movement, climbing, holding food, eating and social interactions scores of MPTP+CQ group were significantly decreased (P<0.05 or P<0.01) when compared with pre-treatment (Supplementary Figure 1). Although the clinical scores of MPTP group decreased slightly during the same period, there was no statistic difference at the end of the treatment period when compared with pre-treatment (Figure 2A, 2B). The clinical score of control group was always “0” during all the period. Meanwhile, some other evaluation items used to evaluate non-motor symptoms were performed before and at the end of the medication. The results showed that the interactivity after stimulation and defense reaction scores of MPTP+LD group were significantly decreased when compared with pre-treatment (25w)(P<0.05) (Figure 2E, 2G), grooming, checking behavior, interactivity after stimulation scores and spontaneous interactivity of MPTP+CQ group were significantly decreased (P<0.05 or P<0.01) when compared with pre-treatment (25w) (Figure 2C–2F). There is no any significant change of hypersomnia defense reaction, facial expression, and vocalizing scores between each group when compared with pre-treatment (25w) (Figure 2H–2J). In addition, constipation displayed with dry and hard feces after MPTP intoxication, was relieved by both LD and CQ treatment (Figure 2K).

The behavior scores evaluated during the treatment period indicated that CQ could mitigate the motor and non-motor deficits after MPTP intoxication

### CQ improved pathology of monkey models after MPTP intoxication

**Figure 3:**
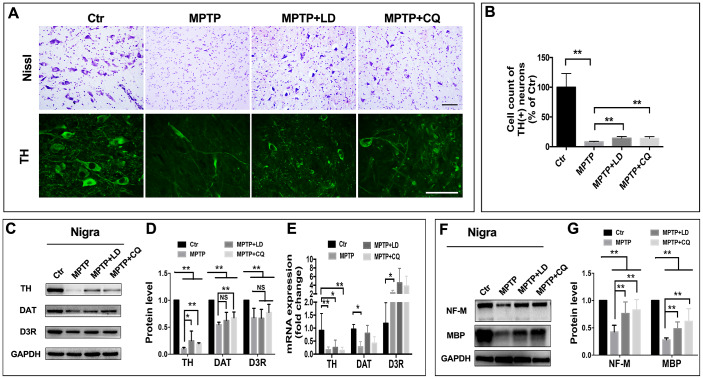
**CQ improved the pathology of monkeys in the SN after MPTP intoxication.** (**A**) Representative images of Nissl and immunofluorescence staining of TH (biomarkers of DA neurons) showed cell loss and cell atrophy in the SN after long-term of MPTP injection, and which were mitigated by both LD and CQ treatment. (**B**) Stereology analysis of cell count of DA neurons in the SN. (**C**, **D**) Western bolts and quantification showed increased protein expression of TH, DAT, and D3R in the SN of MPTP+LD and MPTP+CQ group in comparison to MPTP group. (**E**) Quantification showed the mRNA level of TH, DAT, and D3R in each group. (**F, G**) Western bolts and quantification showed increased protein expression of NF-M and MBP in the SN of MPTP+LD and MPTP+CQ group in comparison to MPTP group. Data expressed as the mean ± SD. *P<0.05, **P<0.01, indicate significant difference. Scale bar=200μm.

In order to determine if the mitigated motor and non-motor deficits were based on the improvement of pathology, histopathological examination was done. Tissue analysis using Nissl staining and immunohistostaining of TH (biomarker of DA neurons) showed that while DA neurons in the SN of MPTP-injected monkeys were atrophied and obviously reduced, more DA neurons were observed after both LD and CQ treatment (Figure 3A). Quantitative stereology demonstrated a significant decrease in cell count of TH(+) cells after the long-term (22 weeks) of MPTP injection, and which was significantly reversed by both LD and CQ treatment (P<0.01) (Figure 3B). Meanwhile, after the intoxication of MPTP, the expression of dopamine synthesis and transport related proteins TH, DAT and D3R (tested by WB) in the SN (Figure 3C, 3D) and striatum (Supplementary Figure 2C–2F) were decreased, and were reversed by both LD and CQ treatment. In addition, the injection of MPTP suppressed the protein levels of NF-M and MBP in the SN (Figure 3F, 3G) and striatum (Supplementary Figure 2G–2I), and which were reversed by both LD and CQ treatment. The results indicated that CQ is involved in the improvement of PD pathology.

### CQ decreased iron content in the SN and suppressed oxidative stress both in vivo and in vitro

**Figure 4:**
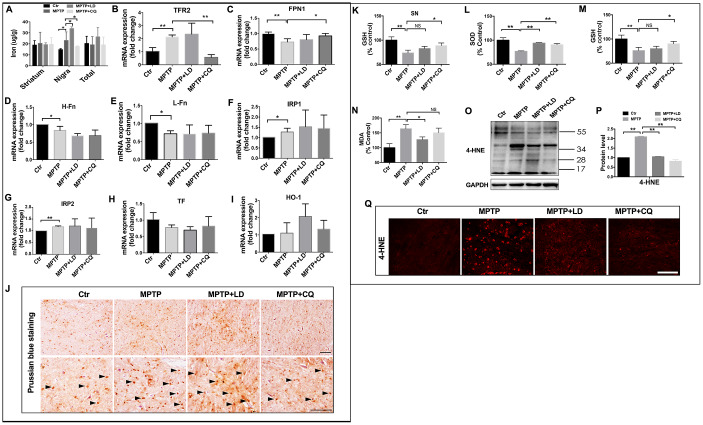
**CQ suppressed iron content and oxidative stress in the SN after MPTP intoxication.** (**A**) Iron content in the striatum, SN and organized in overall (total) of each group tested by flame atomic absorption spectrometry in each group. (**B**–**I**) Quantification showed the mRNA level of iron metabolism related genes, including TFR2, FPN1, H-Fn, L-Fn, IRP1, IRP2, TF, and HO-1 in each group, respectively. (**J**) Representative images of Prussian blue staining showed iron distribution in the SN of each group. (**K**) Quantification showed GSH levels in the SN in each group, respectively. (**L**–**N**) Quantification showed SOD, GSH, and MDA levels in serum of each group, respectively. (**O**–**P**) Western bolt and quantification showed increased 4-HNE expression after MPTP intoxication, and which was decreased by both LD and CQ treatment. (**Q**) Representative images of immunofluorescence staining of 4-HNE in each group. Data expressed as the mean ± SD. *P<0.05, **P<0.01, indicate significant difference. Scale bar=200μm.

CQ acts as an iron chelator. Here, we found that it selectively decreased iron content in the SN, but no such changes were detected in striatum or in total (organized by overall) (Figure 4A). The specific deletion of iron by CQ was also confirmed by Prussian blue staining, which indicated that the increased positive signals after MPTP intoxication was suppressed by 4 weeks of CQ addition (Figure 4J). Meanwhile, the decreased iron transport, as indicated by the downregulated expression of iron uptake transporter TFR2 (Figure 4B), and increased iron efflux, as indicated by the upregulated expression of iron efflux transporter FPN1 (Figure 4C), may be responsible for the deletion of iron in the brain after CQ treatment. However, the other iron metabolism-related genes (including H-Fn, L-Fn, IRP1, IRP2, TF, and HO-1), were likely not responsible for the CQ treatment, although H-Fn, L-Fn, IRP1 and IRP2 were significantly decreased after the MPTP intoxication.

Iron, as a transition metal, is capable of intensify oxidative stress and increase reactive oxygen species (ROS). To explore whether oxidative stress was inhibited by CQ treatment, oxidative damage was analyzed in the SN. 4-HNE, an important product of lipid peroxidation, was evaluated by both immunofluorescence staining and western blot. A widespread increase of 4-HNE positive signals was observed in the SN after MPTP intoxication, while which was suppressed by both LD and CQ treatment (Figure 4Q). The same result was confirmed by Western blot test (Figure 4O, 4P). This decreased 4-HNE level in the SN after addition of CQ was also accompanied by the increase of GSH in the SN (Figure 4K) (which was similar with Kaur’s study [29]), as well as the increase of SOD and GSH activity and the reduction of MDA level in serum (Figure 4L–4N) (which could reflect the severity of brain lesions to a certain degree [33, 34]).

**Figure 5:**
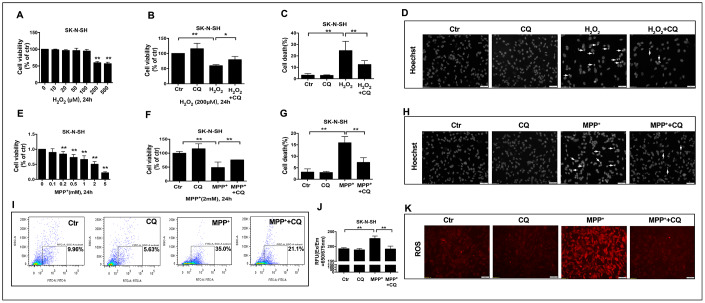
**CQ attenuated MPTP toxicity by decreasing ROS level *in vitro*.** (**A**, **E**) Cell viability was determined for SK-N-SH cells treated with different concentration of H_2_O_2_ (0, 10, 20, 50, 100, 200, 500μM) or MPP^+^ (0, 0.1, 0.2, 0.5, 1, 2, 5mM) for 24h, respectively. (**B**, **F**) Low dose of CQ (5μM) increased cell viability in the absence or presence of H_2_O_2_ (200μM) or MPP^+^(2mM) tested by CCK-8 kit, respectively. (**C**, **G**) CQ decreased cell death tested by Hoechst staining after the exposure of H_2_O_2_ (200μM) or MPP^+^(2mM) for 24h, respectively. (**D**, **H**) Representative images of Hoechst staining for SK-N-SH cells treated with H_2_O_2_ (200μM) or MPP^+^(2mM), respectively. (**I**–**K**) CQ decreased ROS in the absence or presence of MPP^+^(2mM) tested by flow cytometry, microplate reader, and microscope, respectively. Data expressed as the mean ± SD. *P<0.05, **P<0.01, indicate significant difference. Scale bar=50μm.

Subsequently, to explore whether the role of CQ in inhibiting oxidative stress was dependent on the removal of iron or the direct antioxidant effects, SK-N-SH cells were used. We found that when applied alone at low dose (5μM), CQ could increase the cell viability (Figure 5B, 5F), and it could reverse cell death caused by MPP^+^ (an active metabolite of MPTP) or H_2_O_2_ (Figure 5B, 5C, 5F, 5G). Meanwhile, CQ could also suppress the apoptosis (Figure 5D, 5H) caused by exposure of H_2_O_2_ or MPP^+^, as well as ROS caused by exposure of MPP^+^ tested by both flow cytometry and fluorescence microscope. (Figure 5I–5K).

These findings suggested that the efficacy of CQ may be dependent on its chelation of iron and direct inhibition of oxidative stress.

### CQ activated AKT /mTOR pathway in both *in vivo* and *in vitro* system

**Figure 6:**
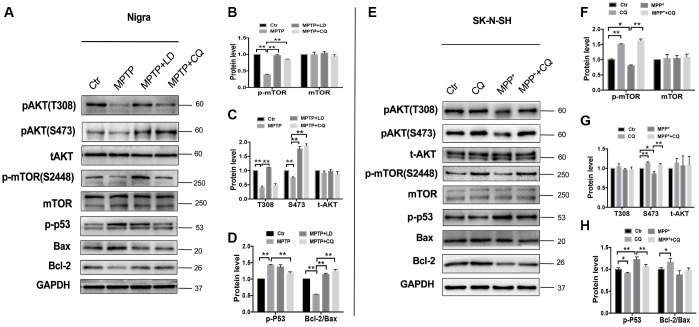
**CQ play neuroprotective effect by improving AKT/mTOR survival pathway and blocking p53 medicated cell death.** (**A**–**D**) Western bolts and quantification showed decreased expression of phosphor-AKT (S473 and T308), phosphor-mTOR (S2448) and Bcl-2, as well as increased p-p53 and Bax in the SN of monkeys, and which were reversed by both LD and CQ treatment. (**E**–**H**) Western bolts and quantification showed decreased expression of phosphor-AKT (S473 and T308), phosphor-mTOR (S2448) and Bcl-2, as well as increased p-p53 and Bax in SK-N-SH cells, and which were reversed by CQ treatment. Data expressed as the mean ± SD. *P<0.05, **P<0.01, indicate significant difference.

It is well known that AKT /mTOR pathway controls cellular growth, proliferation and survival. Here, we shown that MPTP exposure decreased phosphor- AKT(S473 and T308) activity in monkey model, indicated by increased phosphor-p53 and Bax protein levels, and decreased Bcl-2 protein level, and this induction occurred concurrently with the inhibition of phosphor- mTOR. Remarkably, this could be reversed by 4 weeks of CQ treatment (Figure 6A–6D). Furthermore, such changes were confirmed by in SK-N-SH cells. Briefly, we observed a suppression of phosphor-mTOR, phosphor-AKT(S473 and T308), phosphor-p53 and Bcl-2/Bax in MPP^+^ treated SK-N-SH cells, and which were strongly reversed by CQ treatment added 15min later than MPP^+^(Figure 6E–6H). These results displayed the activation function of CQ on AKT/mTOR signals.

## DISCUSSION

Previous studies detected that PD monkey models showed spontaneous recovery of motor symptoms and variable severity of symptoms after the same protocol of MPTP exposure [15–17, 35]. So we carried out a long-term, chronic and progressive MPTP injection schedule according to individual sensitivity. After 22 weeks we generated stabilized monkey models of PD displaying with classic motor symptoms (especially resting tremor) and non-motor symptoms (including salivation, dysphagia, lethargy, less vocalization, constipation, mask face, and hyporeactive social interactions). The classic and stabilized motor and non-motor symptoms should be resulting from the more than 90% DA neurons loss in the SN, as well as dopamine deficiency in striatum without any TH-positive terminals (data not shown). Spontaneous recovery of motor symptoms probably results from neuronal loss (70%) in the SNpc [36, 37]. Non-motor deficits are quite common in PD patients, and have gained relevance due to their impact on patients quality of life in recent years. In contrast with motor symptoms, which respond to dopaminergic medication, non-motor deficits are often poorly recognized [38]. Interesting, this discovery is further confirmed by the present study, and we found that non-motor deficits respond poorly to LD and better to CQ treatment. Thus indicating that CQ probably be more effective than LD in the treatment of non- motor deficits, and the monkey model in our study, which recapitulated almost all the motor and non-motor symptoms of PD, can be used to further study the pathogenesis and therapeutic strategies of PD. However, neither Lewy bodies, another important pathological feature of PD [39, 40], nor increased a-synuclein (the major component of Lewy body) were detected in the present models (data not shown), likely due to less than 10% of the remaining TH(+) neurons in the SN.

Iron has been shown to selectively elevated in both PD patients and animal models [41–43]. Our previous study found that the degree of iron accumulation was correlated with the disease severity of PD in monkey model [35]. Subsequently, we found that iron overload resulting from oral administration of ferric citrate led to the accumulation of iron in the brain and the appearance of parkinsonism phenotypes [43]. These results indicated that excess iron in the brain indeed plays a role in the pathogenesis of PD. The relationship between excess iron and PD as well as some other neurodegenerative diseases has been debated for decades. Iron, as an essential micronutrient in the nervous system also poses toxicity, since its interconversion between ferrous Fe^2+^ and ferric Fe^3+^ generates free radicals, which can stimulate iron released from its storage proteins such as ferritin and then further feed ROS [44]. As such, the interplay of iron and oxidative stress is likely involved in a feed forward vicious cycle, continuously causing cellular damage and leading to neurodegeneration. Specifically, iron could also induce DA neurons death in PD by some other mechanisms, such as causing neuromelanin (an effective endogenous metal chelator and could convert DA into a stable compound in DA neurons) overload, which leads to oxidation of DA to DA-o-quinone (a highly reactive molecule with known toxicity [45] and could promote the aggregation of a-synuclein [46]) and 6-OHDA (a minor byproduct of iron-mediated DA oxidation [47] and a neurotoxin commonly used to produce PD animal model), stimulating the aggregation of a-synuclein by direct binding [48], and enhancing the oxidation of MPTP (one of the popular compound used for inducing animal model of PD) to MPP^+^ in medium without cells [49]. Furthermore, some recent studies found that ferroptosis, which is a new iron-dependent and regulated process of cell death that involves in depletion of GSH and an increase of lipid peroxidation [50], is also involved in the pathogenesis of PD [51, 52], such as, Do van’s study [53] found that MPTP could induce DA neurons death by ferroptosis pathway, while which could be reversed by both iron chelator (deferiprone) and its inhibitor (such as ferrostatin 1). Actually, before the discovery of ferroptosis, iron chelators (such as desferrioxamine, VK-28, EGCG, and M30) have been widely used in the treatment of PD model [54–57].

Clioquinol (CQ), another effective metal ion chelating agent, was widely used as an antiparasitic agent at early stage, and then gradually withdrawn from the market due to high dosage (1500 mg/d) causing subacute optic nerve inflammation (SMON) (Asao, 1979; Osterman, 1971). In recent years, it has been utilized in clinical phase I for the treatment of hematological malignancies, clinical phase III for the treatment of dermatitis and eczema, and clinical phase II for the treatment of AD [58]. Because of its ability to freely cross the blood-brain barrier to chelate of metal ions (iron, copper, zinc) and reduce the toxicity in neurodegenerative diseases, CQ is being gradually used as a neuroprotective candidate for neurodegenerative disorders by researchers [25, 26]. The mechanism involved in these findings may be redox-silencing of reactive Fe^2+^, while the underling mechanism responsible for the protective effect is not well understood. In the present study, we noticed that at the end of the experiment, iron content in the SN of MPTP group monkeys statistically increased when compared with control group, and which was accompanied by increased levels of oxidative damage and DA neurons death. While addition of CQ could not only act as an iron chelator decreasing the iron content in the SN to the normal levels without any obvious side effects on body weight, blood routine, and live and kidney structure (Supplementary Figure 2A, 2B), which was consisted with Kaur and Yassin’s finding that CQ can reduce bioavailable brain iron in normal mice with no apparent adverse health and behavior effects [29, 59], but also act as an antioxidant directly resisted oxidative stress, resulting in the improvement of pathological defects and motor and non-motor deficits.

These *in vivo* results were in good agreement with our parallel *in vitro* studies. In the cell culture study, we noted that low dose (5uM) of CQ, when applied alone did not cause or exacerbate cell death, and it could increase the cell viability caused by both MPP^+^ and H_2_O_2_ exposure, respectively. So it is clear that low dose of CQ could directly suppress oxidative stress. Interestingly, the results were consistent with Filiz’s study [60]. The study noted that CQ (25μM) could protect BE(2)-M17 human neuroblastoma cells against H_2_O_2_ toxicity, while which can’t be observed by a number of hydrophilic and hydrophobic metal ligands (including 8-hydroxyquinoline (8-HQ), neocuproine, (NC), bathocuproine sulphonate (BCS), bathophenanthroline sulphonate (BPS)). So the results strongly suggested that the protective role of CQ in PD is not simple because of its chelating effect on iron which induces ROS, but also because of its antioxidant function which is at least partially due to its phenol hydroxyl structure directly reacting with hydrogen peroxide. Meanwhile, the results together with previous studies [53, 61] also suggested that ferroptosis probably be an important cell death pathway of DA neurons, and iron chelator, especially CQ, may be strong drug candidates to pharmacologically modulate the ferroptosis signal pathway.

**Figure 7:**
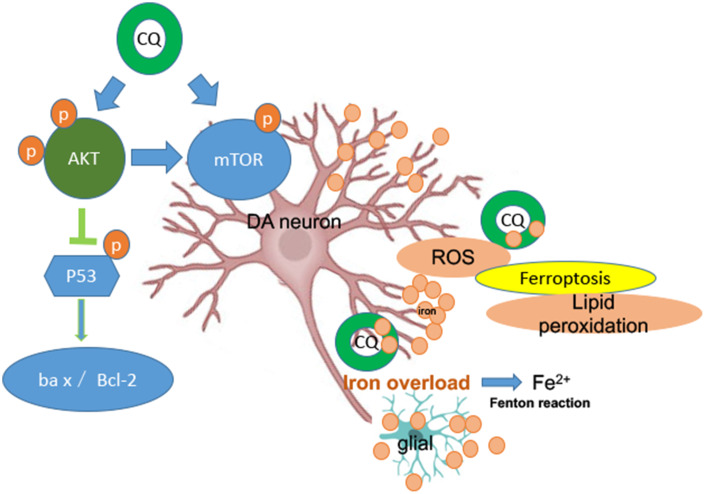
**A summary diagram is shown. In the brain both glial and DA neurons are involved with iron dysfunction during the development of PD.** Specifically, both increased levels of ROS and lipid peroxidation (main features of ferroptosis) were happened in the MPTP-induced monkey model, while which could be reversed by low dosage of CQ treatment. So ferroptosis dysfunction probably be involved in the pathogenesis of PD. Meanwhile, the protection effect of CQ was depending on the activation of the AKT/mTOR survival pathway and the prevention of p53-medicated cell death.

It is well known that AKT/mTOR pathway plays a key role in numerous cellular functions, such as proliferation, protein synthesis and cell growth. A growing number studies have already noted that AKT/mTOR pathway is implicated in the process of neurodegenerative disease [62, 63], such as, mTOR pathway plays a critical role in the regeneration and synaptic plasticity after central nervous system injury [64]. Meanwhile, AKT/mTOR pathway is also implicated in the iron metabolism. Such as, AKT and mTOR activities are involved in regulating cellular access to extracellular nutrients (such as iron) [65, 66]. Here, we found that MPTP exposure downregulated the protein levels of p-AKT and p-mTOR in the SN of PD monkeys and in SK-N-SH cell system, and could be significantly reversed by CQ treatment. Meanwhile, we also found that CQ could down-regulate p-p53 activity in both monkey models and cell systems, accompanied by increased Bcl-2/Bax protein levels. Abnormal p53 activation has been shown in some forms of neurodegenerative disease, such as AD, PD and prion disease [67], and also involved in apoptosis and oxidative stress. Thus CQ could protect cells against p53-mediated apoptosis. These results taken together suggest that CQ can decrease the excessive iron in the SN to normal level and directly protect DA neurons against oxidative stress probably by activating the AKT/mTOR survival pathway and blocking p53-medicated cell death (Figure 7).

## MATERIALS AND METHODS

### Animals and MPTP treatment

All the experimental protocols were reviewed and approved by the Animal Welfare and Use Committee, and all the experimental procedures were conformity with the guidance of the National Institutes of Health Guide for the Care and Use of Laboratory Animal of the United States. In total, twelve healthy adult rhesus monkeys (Macaca mulatta lasiotis, aged 4-5 years, and weighed 3.5-5 kg at the start of the study) were obtained from Sichuan Primed Biological Technology Co., Ltd (National Experimental Macaque Reproduce Laboratory) (Certificate No SCXK (Chuan): 2013-105). Two weeks prior to the experiment, the animals were transferred from their home room to stainless steel monkey cages, one animal per cage, in a feeding room with controlled conditions of temperature (19 to 26°C), humidity (40 to 70%) and light (12 h day and night cycles, lights on 8:00 am). Tap water was provided ad libitum via an automatic bubbler. A standard diet (containing 18% protein, 69% carbohydrates, 3% fat, and 10% water) was fed twice daily. Meanwhile, vegetables and fruits with equal nutrients were provided to each animal every day. The healthcare and maintenance of non-human primates were performed under the supervision of specialty veterinarians. All biohazard waste was autoclaved before disposal.

Monkeys were randomly divided into two groups: normal (control) group (n=3) and MPTP group (n=9). Monkeys from MPTP group were administered with MPTP (MPTP-HCl, Product Number: M0896, Sigma, St. Louis, MO) by intramuscular injection daily at the beginning of the study, and then the MPTP dose was gradually added to 0.5mg/kg at the end of the experiment. Monkeys from control group were injected with saline instead, and the other conditions were the same with MPTP group. Specifically, at the beginning of the experiment, all monkeys received the same and minimize dosage (0.2mg/kg) of MPTP, when one third of monkeys showed obvious motor deficits, the remained monkeys with no obvious phenotype will receive an increased dosage (0.3mg/kg) of MPTP daily, and when two third of monkeys showed classic motor deficits, the remained monkeys showed no obvious phenotype of PD will receive another increased dosage (0.4mg/kg or 0.5mg/kg) of MPTP daily. Meanwhile, during the progression, MPTP should be withdrawn when monkeys displayed with dysphagia and severely impeded feeding, otherwise the monkeys showed classic motor deficits will proceed the MPTP injection every 3 to 4 days to maintain the behavior phenotypes. To prevent the spontaneous recovery of motor symptoms, all the monkeys were proceeded with IV injection of MPTP at their own maximum dosage during the last week (22^th^ week). Overall, the outline of the protocol was showed in Figure 1A.

### Clinical motor evaluation

Monkeys were monitored by video surveillance with no observers in the room for recording the spontaneous activity during all the procedures. Parkinsonian severity was analyzed by three examiners familiar with the scoring rules of Kurlan rating scale [68] in MPTP-injection period. Briefly, the Kurlan rating scale including facial expression(0-3), resting tremor (0-3), action or intention tremor (0-3), posture (0-2), gait (0-3), bradykinesia (generalized) (0-4), balance/ coordination (0-3), gross motor skills (upper limb) (left/ right) (0-3), gross motor skills (lower limb) ((left/ right) (0-3), defense reaction (0-2). Total score can vary from 0 to 29. Occasionally, a score of 0.25 was given to reflect very mild motor abnormalities. The score represented the maximal (range of movement) or typical (posture, bradykinesia) behavior in the defined time period. Increasing levels of parkinsonism were reflected by an increased range of the scores. The spontaneous activity was assessed by the average score of the week.

### Clioquinol treatment

Three weeks after the last MPTP injection, when all MPTP-injected monkeys showed with stabled and classic PD symptoms, CQ treatment was carried out twice daily for four weeks, and L-dopamine (LD) was used as positive treatment at the same time. Briefly, MPTP group monkeys were randomly divided into three subgroups: MPTP group (n=3), MPTP+CQ group (n=3) and MPTP+LD group (n=3). Both CQ (Sigma, 10mg/kg) and LD (Sigma, LD/carbidopa= 12:3 mg/kg) were administered twice daily by mixed with 10 mg of standard diet, respectively. MPTP group monkeys with no any treatment were accepted the same dose of 10mg standard diet.

### Response assessments

According to Imbert’s analysis [69], Kurlan rating scale was a working basis to dispose of clinical rating scale, and Papa scale is more sensitive to reflect the treatment effect, so we selected Papa scale to evaluate behavior improvement after medication. Qualitative response assessments of CQ treatment were performed by Papa scale [70]. Briefly, the Papa scale including: posture (0–2); gait (0–2); tremor (0–2); general mobility (0–4), hand movements (0–2), climbing (0–4), holding food (0–1), eating (0–1), social interaction (0–2). Occasionally, a score of 0.25 was given to reflect very mild motor abnormalities. Total score can range from 0 to 20. Additionally, non-motor symptoms (especially mental disorders) were also evaluated by constipation, hypersomnia (0= normal; 1= hypersomnia), facial expression (0= normal; 1= slight, but noticeable diminution of facial expression; 2= moderate hypomimia; 3= severe, masked/fixed facies) [68], grooming (0= present; 1=absent), checking behavior (0= present, looking around, observant; 1= absent) [71], interactivity after stimulation (0= present; 1= absent) [72], spontaneous interactivity (0= present; 1= absent), defense reaction (0= Normal; reacts appropriately to examiner (steps back, opens mouth to show teeth, etc.); 1= Detectably impaired, slowed or abnormal defensive response; 2= little to no response upon good provocation) and vocalizing (0= present;1= absent), after CQ treatment at the same time.

### Tissue collection

At the end of the experiment, animals were sacrificed under deep anesthesia by overdose of sodium pentobarbital (50 mg/kg, i.v, with effect confirmed by absence of corneal reflex), and the brains were rapidly removed on ice. The left cerebral hemisphere was divided into coronal slabs of 4 mm thickness and then fixed in 4% paraformaldehyde phosphate buffer solution (pH 7.4) for further use, meanwhile brain tissue of the SN from right cerebral hemisphere was stored in liquid nitrogen until use.

### Histopathology

Brain slabs were cryoprotected by soaking in 20 and 30% sucrose solution at 4°C and sliced into 30μm coronal sections. Firstly, sections were floated in 0.1% chrome alum-gelatin solution and mounted on glass slides. After drying at room temperature, sections were processed for Nissl staining. Then standard immunohistochemistry was carried out to examine DA neurons damage using anti-TH antibody (ENZO, USA) and lipid peroxidation using anti-4-HNE (Abcam, USA).

### Western blotting

Standard western blotting procedures were carried out with the flowing antibodies: anti-TH antibody (ENZO, USA), anti-dopamine transporter (DAT) antibody (Bioss, China), anti-dopamine D3 receptor (D3R) antibody (Bioss, China), anti-NFM antibody (Abcam, USA), anti-MBP antibody (Abcam, USA), anti- phospho-P53 (Beyotime, China), anti-Bax antibody (Boster, China), anti-Bcl-2 antibody (Boster, China), anti-GFAP antibody (Millipore, USA), anti-phospho-mTOR (Ser2448) antibody (CST, USA), anti-mTOR antibody (Bioss, China), anti-phospho-AKT(S473) antibody (CST, USA), anti-phospho-AKT(T308) antibody (CST, USA), anti-AKT antibody (CST, USA), anti-4-HNE (Abcam, USA), anti-GAPDH (Boster, China), anti-β-actin (Boster, China).

### Quantitative realtime PCR

Total RNA was extracted from the SN using Trizol reagent (Takara, Japan), then subjected to reverse transcription using the Prime Script RT reagent kit (Takara, Japan) and store at -20°C. Quantitative Real-time PCR was performed using Bio-Rad CFX96 system, and the relative gene expression was quantified to internal control as β-actin. Primer sequences of target genes was shown in Table 2.

**Table 2:** Gene and primer information.

**Gene**	**Sequences (5’-3’)**	
TH	F: GCAGTTCTCCCAGGACATCG	R: TCAGACAGGCAGTGCAGTAGCT
DAT	F: CTCATCTCGCTGTATGTCGGC	R: GGCTGTTCCAGGAGTTGTTGC
DRD3	F: TGTCCTTCTACTTGCCCTTTGG	R: TGACACTGTTGCACTGACTGTTCTG
H-Fn	F: CCAGAACTACCACCAGGACTCAG	R: AGTTTCTCGGCATGTTCCCTT
L-Fn	F: TTCATGCCCTGGGTTCTGC	R: TGTTGAGGTTGGTCAGGTGGTC
Tf	F: CCAACAACAAAGAGGGATACTACG	R: GGAACCATCAAGGCACAGCA
TfR2	F: GAAGCTGCGGCAGGAGATCTA	R: GCGACACGTACTGGGAAAGGA
HO-1	F: GCCAGTGCCACCAAGTTCAA	R: CGGTCTTGGCCTCTTCTATCAC
IRP1	F: TGAGAAAGAGCCATTGGGAGTAA	R: ATGACATACTGACGCTCCACTGC
IRP2	F: AGAGACTGGGCTGCCAAAGG	R: GGAGCTATGCCAATTCCAATCA
FPN1	F: AATTCTGCTAATATTGTCCCGGAG	R: TCAAAGGACCAAAGACCGATTC
β-actin	F: GTGACGTGGACATCCGTAAAGAC	R: CAGAGTACTTGCGCTCAGGAGG

### Iron content tested by flame atomic absorption spectrometry

Iron content in the striatum and SN were quantified by flame atomic absorption spectrometry as descripted by our [43] previous study. Briefly, samples were digested in concentrated nitric acid at 180°C for 2h, (Mars 6, Thermo Fisher Scientific Inc. Waltham, MA, USA). Then, the iron concentration was determined by flame atomic absorption spectrometry (Model PE-800, PerkinElmer, USA). Validation of the mineral analysis was conducted using green tea or bovine liver powder as a standard reference material (National Institute of Standards and Technology, Beijing, China).

### Prussian blue staining

As previous described [43], after mounted on glass slides, the slices were incubated in Prussian blue staining solution containing 7% potassium ferrocyanide and 3% hydrochloric acid at a 1:1 ratio for 30min, followed by three washes in PBS. Subsequently, the slices were soaked in 1% H_2_O_2_ for 30 min and washed 3 times with distilled water. Finally, the slices were incubated in distilled water containing 0.25 mg/mL DAB and 0.02% H_2_O_2_ for 10min, counterstained for 5 min, dehydrated with gradient alcohol and mounted with xylene.

### Biochemical reaction assay

As previous described [34], the activity of superoxide dismutase (SOD) and the levels of glutathione (GSH) and malondialdehyde (MDA) were determined by a biochemical reaction assay kit (Nanjing Built Biology, Nanjing, China) according to the manufacturer’s instructions.

### Cell cultures

SK-N-SH cell line was used in this study (both purchased from Kunming Cell Bank of Chinese Academy of Sciences, China) as they have been used in previous similar studies [35, 73]. Cells were routinely cultured in DMEM (Gibco, Waltham, MA, USA) supplemented with 10% FBS and 1% penicillin/ streptomycin, and maintained at 37°C in a humidified atmosphere of 5% CO_2_. Twenty-four hours after plating or when the cell density was 60%-70%, the cells were immediately pretreated with MPP^+^ (Sigma, USA) or H_2_O_2_, then co-treated with clioquinol (CQ) (Sigma, USA) for another hours.

### Cell viability assay

SK-N-SH cells were plated in 96-well plates, respectively. After treatment, cell viability was measured by Cell Counting Kit-8 (CCK-8) system according to the instruction provided by the manufacturer (Dojindo, CK04-11, Japan). Briefly, CCK-8 solution (10μL per 100μL of medium in each well) was added in each well, and the plates were then incubated at 37 °C for 1 h. The absorbance of each well was read at 450 nm using a microplate reader (Thermo, USA). Each experiment was repeated four times.

### Evaluation of cell death

Apoptotic nuclei were detected by Hoechst (Beyotime, China) staining. Briefly, after treatment, cell death was quantified and analyzed as previous described [7].

### Measurement of reactive oxygen species (ROS) production

Measurement of ROS was performed in SK-N-SH cells. Briefly, after treatment rosup as ROS positive reagent was treated for 20 min, then DCFH-DA probe was added in the medium, and the following procedures were performed according the manufacturer’s instruction (Abcam, US). The measurement of fluorescence was performed by flow cytometer (BD, FACSCalibur, USA), and normalized by control wells. Meanwhile, ROS (Deep Red Fluorescence) (Abcam, US) were also detected by photograph with fluorescent microscope and microplate reader. Each treatment was repeated in triplicate.

### Statistical analysis

Data represent the mean and standard deviation (x ± SD). Student’s t-test was used to determine the difference between two experimental groups. One-way analysis of variance (ANOVA) and Dunnett’s post hoc test were used for multiple comparisons between more than two groups. *p<0.05, **p<0.01.

## Supplementary Material

Supplementary Figures
