# Supplementary material for: Clioquinol improves motor and non-motor deficits in MPTP-induced monkey model of Parkinson’s disease through AKT/mTOR pathway
Source: Aging (Albany NY). 2020 May 18;12(10):9515–33. doi: 10.18632/aging.103225 (PMC7288933; doi:10.18632/aging.103225)
Supplement: Supplementary Figures [file aging-12-103225-s001..pdf]

SUPPLEMENTARY FIGURES

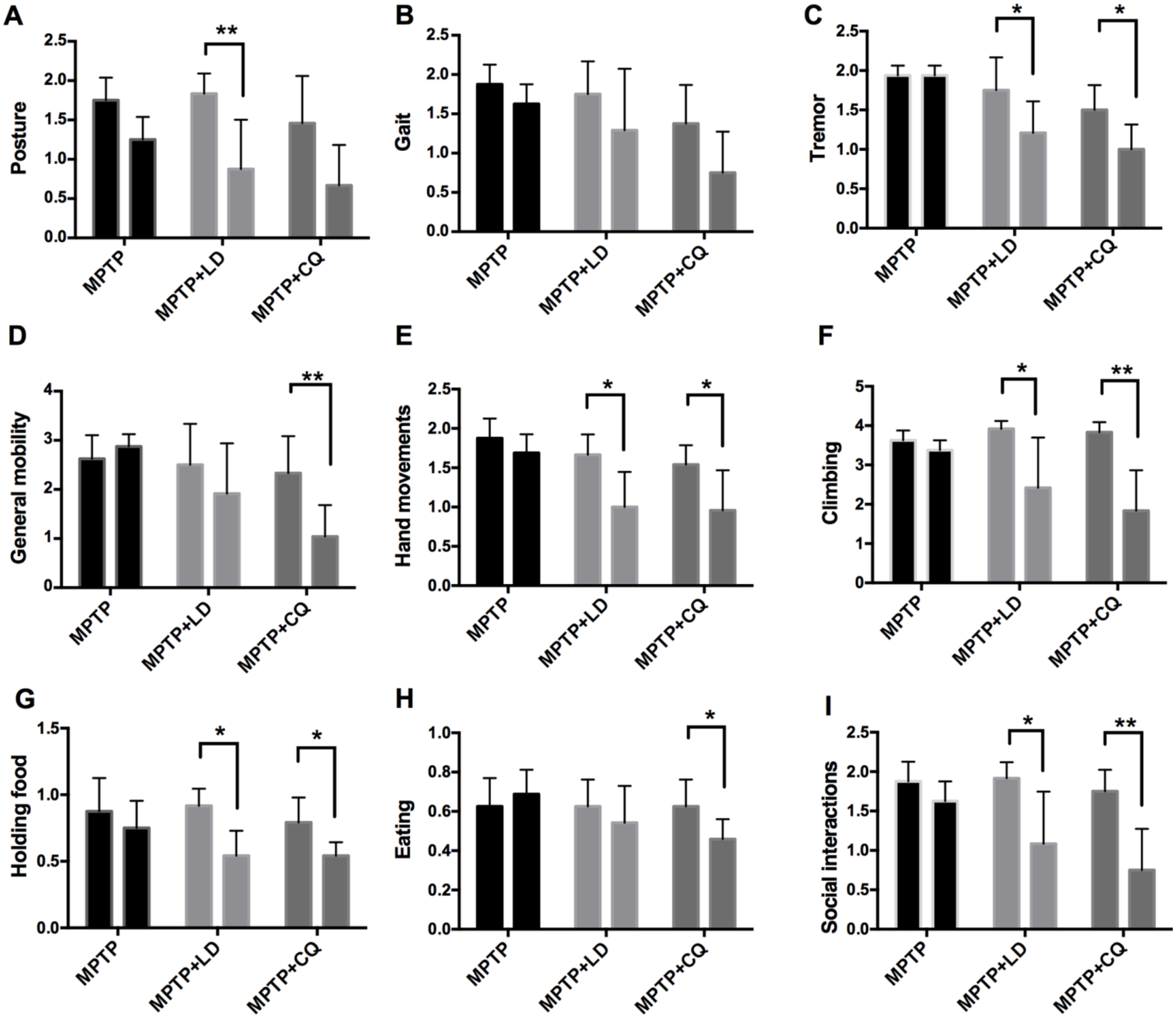

Supplementary Figure 1. Comparison of each item of Papa scale before and after treatment. L-dopamine (12mg/kg) and CQ (10mg/kg) were orally administrated for 4 weeks after MPTP intoxication. Both LD and CQ treatment significantly reduced the tremor score(**C**), hand movements score(**E**), climbing score(**F**), holding food score(**G**) and social interactions score(**I**). Additionally, LD significantly reduced posture score(**A**), and CQ significantly reduced gait score(**B**), general mobility score(**D**) and eating score(**H**). Data expressed as mean ± SD. *P<0.05, **P<0.01, indicate significant difference.

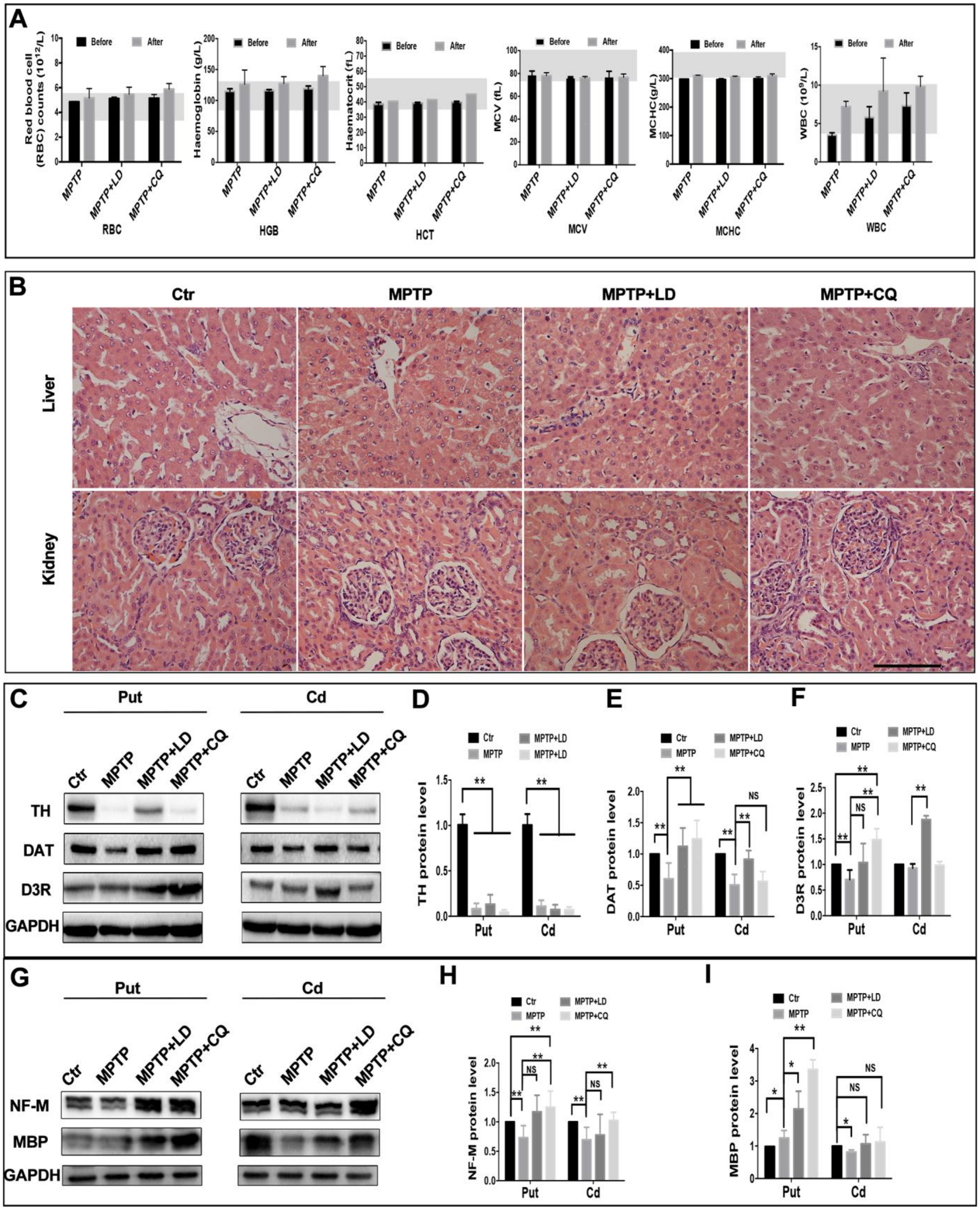

Supplementary Figure 2. CQ caused no side effect on peripheral blood, liver and kidney and CQ improved the pathology in the Striatum. (**A**) Quantification showed the blood biochemistry test results were all in the normal level before and after CQ treatment. Abbreviation: RBC: Red blood cell counts; HGB: Haemoglobin; HCT: Haematocrit; MCV: Mean corpuscular volume; MCHC: Mean corpuscular haemoglobin concentration; WBC: White blood cell counts. (**B**) Representative images of hematoxylin and eosin staining showed normal architecture in liver and kidney from each group monkeys. Scale bar=200μm. (**C**–**F**) Western bolts and quantification showed increased expression of TH, DAT, and D3R in the striatum of t MPTP+LD and MPTP+CQ group monkeys in comparison to the MPTP group. (**G**–**I**) Western bolts and quantification showed increased expression of NF-M and MBP in the striatum of the MPTP+LD and MPTP+CQ group monkeys in comparison to the MPTP group. Data expressed as mean ± SD. *P<0.05, **P<0.01, indicate significant difference.
